# Monitoring Bivalve Behavior and Physiology in the Laboratory and Field Using Open-Source Tools

**DOI:** 10.1093/icb/icac046

**Published:** 2022-05-20

**Authors:** Luke P Miller

**Affiliations:** Coastal and Marine Institute and Department of Biology, San Diego State University, San Diego, CA 92182, USA

## Abstract

Bivalve molluscs have been the focus of behavioral and physiological studies for over a century, due in part to the relative ease with which their traits can be observed. The author reviews historical methods for monitoring behavior and physiology in bivalves, and how modern methods with electronic sensors can allow for a number of parameters to be measured in a variety of conditions using low-cost components and open-source tools. Open-source hardware and software tools can allow researchers to design and build custom monitoring systems to sample organismal processes and the environment, systems that can be tailored to the particular needs of a research program. The ability to leverage shared hardware and software can streamline the development process, providing greater flexibility to researchers looking to expand the number of traits they can measure, the frequency and duration of sampling, and the number of replicate devices they can afford to deploy.

## Introduction

Bivalves can be important foundation species and ecosystem engineers in a variety of marine and freshwater habitats, providing hard substrate and three-dimensional structure that can be utilized by a variety of other species (Stephenson and Stephenson 1972; Suchanek 1978; Jackson et al. 2001). As filter feeders and competitors for primary space, bivalves can have substantial effects on planktonic and benthic communities, which, depending on the circumstances, might contribute valuable ecosystem services in their native habitats (Lenihan 1999; Grabowski and Peterson 2007; Grabowski et al. 2012) or substantial disruptions to ecosystems where a bivalve species has been introduced (Hockey and van Erkom Schurink 1992; Seed and Suchanek 1992; Crooks 1998; Pace et al. 1998). A substantial aquaculture industry has developed around several species of sessile bivalves, commonly with animals being settled and raised attached to suspended ropes or other structures, and providing economic value through food production, nutrient remediation, and other associated uses worth billions of dollars worldwide (Shumway et al. 2003; van der Schatte Olivier et al. 2020).

The relatively sessile nature of many bivalves has made them attractive study species for physiological, behavioral, and ecological experimentation, as it can simplify housing animals in laboratory aquaria or monitoring in the field. In addition to basic research, monitoring bivalve behavior and physiology has found applications in water quality monitoring and aquaculture, including efforts such as the Mussel Watch program that has used mussels and oysters as biosentinel organisms for monitoring pollutant accumulation in tissues since the late 1970s (Farrington et al. 1983; Goldberg 1986). The use of bivalves for automated monitoring of changes in water quality conditions at drinking water facilities dates back to the 1990s (de Zwart et al. 1995; Borcherding 2006). Groups of mussels or clams with attached sensors can be monitored for abrupt shell closure events that could mark the introduction of toxic substances in water intake systems. In aquaculture settings, monitoring of parameters such as heart rate and shell opening can give important insight into growing conditions (Andrewartha et al. 2015).

The goal of this review is to illustrate some of the history of those studies and remark on how modern open-source electronics hardware and software have expanded the range of possibilities for further studies of physiology, behavior, and ecology of bivalves, although the general techniques here are applicable to a much broader range of organisms. Importantly, these open-source approaches also have the potential to improve the accessibility of those techniques to a broader audience of investigators through declining costs and increasing customizability, and provide tools that can be used in both basic research and in applied settings such as aquaculture and water quality monitoring.

## Review of past and present methods for monitoring bivalve behavior and physiology with sensor systems

### Valve gaping

The act of opening or closing the paired shell valves (“gaping”) of a bivalve mollusc is an easily observable process with several key implications for the animal. Closed valves might serve to protect the animal from predators or isolate the animal from poor water quality conditions, while valves must be opened to allow respiratory gas exchange, feeding, excretion, spawning, and byssal thread secretion. Researchers have been devising “valvometer” equipment since the early 1900s to track patterns of valve gaping. Marceau (1909) first described the use of a kymograph to track valve gaping through time, by attaching a balanced lever arm to one valve of the animal while a scribe on the opposite end of the lever arm marked a trace on a rotating smoked drum. This simple technique achieved the open-source ideal of being easy to describe and possible to replicate and modify by anyone with access to the appropriate mechanical equipment. Variations on the kymograph valvometer appeared for the next few decades (Galtsoff 1928; Loosanoff and Nomejko 1946), including a version that could be deployed on a dock to measure clams situated on the benthos more than 3 m underwater (Loosanoff 1939). Lever-actuated systems gave way to electrical sensor systems such as strain gages that could be recorded with paper chart recorders (Shumway and Cucci 1987) or later computer data acquisition systems (Porter and Breitburg 2016). Lever-arm-actuated systems and strain-gage systems had the disadvantage of needing to anchor the opposite valve of the animal so that only one valve could be moved to actuate the measurement system, which might require restraining species that might normally move, such as burrowing clams.

The development of electronic measurement systems that could measure the distance between the opposing valves without necessarily requiring one valve to be immobilized allowed more flexibility in experimental design. The simplest such systems rely on magnetic reed switches, which open or close a circuit based on proximity to a magnet (Borcherding 2006; García-March et al. 2008). The magnet can be glued to one valve of the shell, and the waterproofed reed switch glued to the opposite valve. The result is a system that can provide a simple open or closed binary signal that can be recorded on a variety of data logging systems including simple event loggers (UA-003–64, HOBO Pendant Event Data Logger, Onset Computer Corporation, Bourne, MA) or via computer. More sophisticated approaches provide a signal proportional to the distance between the two shell valves rather than the discrete on-off output of a reed switch. Being able to measure the distance between the valves can help distinguish narrow opening events that may not be sufficient for fully extending the siphons and pumping water from wider gape openings that allow normal water exchange (Jou et al. 2013). Distance-measuring methods can include impedance-based measurements between a pair of electrodes attached to the valves (Tran et al. 2003), while most modern approaches utilize an approach that measures the strength of a magnetic field. A pair of electromagnetic coils can be attached to the shell valves, with one coil being powered to produce a magnetic field that induces current to flow in the opposite coil (de Zwart et al. 1995; Schwartzmann et al. 2011; Sow et al. 2011; Jou et al. 2013). Alternatively, a permanent magnet and a Hall effect sensor can be attached to opposite valves of the shell, with the Hall effect sensor producing a voltage change in proportion to the strength of the magnetic field produced by the nearby magnet (Wilson et al. 2005; Robson et al. 2007, 2010a, 2010b; García-March et al. 2016; Miller and Dowd 2017, 2019; Comeau et al. 2018; Lassoued et al. 2019).

### Heart rate

While observing shell valve opening and closing is straightforward, early researchers wishing to directly observe the heart beating *in situ* needed to cut a window in the shell (Segal 1956) or use young individuals with translucent shells (Schlieper 1955). The location of the heart near the inner surface of the shell permitted this type of observation, but more commonly researchers used impedance electrodes inserted through small holes drilled in the shell near the heart (Helm and Trueman 1967; Shick et al. 1986; Nicholson 2002; Braby and Somero 2006). The movement of the heart modified the electrical impedance between the two electrodes, which could be recorded with a chart recorder or computer data acquisition system.

A less invasive method for measuring heart rate using an infrared (IR) LED emitter and detector pair was developed by Depledge and Andersen (1990) for use on crustaceans and molluscs. The method relies on the same technology used in modern medical pulse oximeters (photoplethysmography). IR light is emitted from a LED into the body, and some of that light is reflected back up to toward the sensor, which incorporates a detector tuned to the particular wavelength of light being emitted by the sensor. In the human application, the change in shape of capillary beds in the wearer's finger or toe as blood is pumped through the circulatory system modify the intensity of light sensed by the detector, and the varying signal can be used to estimate the pulse (Allen 2007). Because the calcium carbonate shells of molluscs and crustaceans are semi-transparent to IR light, emitter-detector systems can be attached to the outside of the shell adjacent to the heart and provide non-invasive recordings of heart movement (Depledge and Andersen 1990). IR heart rate sensors have been used on a variety of molluscs in the laboratory (Rovero et al. 1999; Gandra et al. 2015; Seo et al. 2016; Moyen et al. 2019, 2020; Zhang et al. 2021) and in field settings (Bakhmet 2017; Gurr et al. 2018, 2021). IR heart rate sensors have traditionally been built using analog sensors that produce a continuously varying voltage (or current) output (Depledge and Andersen 1990), which require separate amplification and analog-to-digital converter chips (Burnett et al. 2013). The spread of IR and visible light sensing technology for proximity detection or particle detection, as in smoke detectors (Fuhs et al. 1992), has led to the availability of integrated digital IR emitter–detector chips that incorporate the necessary amplification and analog-to-digital signal conversion. Because computers and microcontrollers must store data as digital representations of values, an analog-to-digital converter is required to change the analog voltage signal, for example a signal of 3.3 V, into a digital number representing that 3.3-V value.

### Temperature

Bivalve body temperature can be measured with a variety of sensor types such as thermistors, thermocouples, or platinum resistive temperature detectors (RTDs). For bivalves fully immersed in water, it may often be sufficient to simply measure the surrounding water temperature and assume the animal quickly reaches thermal equilibrium with the water. For intertidal animals, especially those with greater thermal mass, direct measurements may be necessary (Helmuth 2002). Sensors may be placed on the outer shell surface, which may only give an approximation of internal tissue temperatures, or sensors may be inserted into holes drilled in the shell (Lowe 1974; Miller and Dowd 2017; Moyen et al. 2019).

While each of the parameters described above may be interesting in isolation, it is also often the case that measuring two or more parameters at the same time provides a more complete picture of the condition of the animal (gaping or not gaping, high or low heart rate, changing internal temperature) and its responses to external perturbations (Curtis et al. 2000). Modern data logging systems can allow for simultaneous sampling from multiple sensor types (Andrewartha et al. 2015; Gandra et al. 2015; Olabarria et al. 2016; Miller and Dowd 2017). A growing variety of different sensor types, both analog and digital, can make integrating multiple sensor modalities into a single device straightforward, and can provide increased precision, longer battery life, and in many cases lower costs.

### Open-source data acquisition hardware

The earliest studies described above fit the model of open-source tools, in that relatively simple mechanical or electrical devices could be described in a publication, and thus be replicated by users with access to similar equipment. The advent of computer-based data collection systems created something of a regression in the ease of replication and implementation of research methods, particularly when early computers were costly and the software needed to implement data collection routines was proprietary, or at least not easily distributed in the pre-Internet era. In that same time frame, commercial stand-alone data logging systems became prevalent, which allowed researchers to implement monitoring systems that could run without needing to be tethered to a computer. Those commercial options were traditionally closed-source hardware and software, and might offer relatively limited flexibility in programming or adapting new sensor types (Mickley et al. 2018).

The transformation of computers into lower-cost commodity hardware through the 1990s and 2000s, the growth of open-source software tools for developers, and the ability to easily share software and designs over the Internet all created the potential for more widespread accessibility for researchers, and laid the groundwork for modern open-source hardware and software solutions.

At present, a fully functional Internet-ready computer (sans keyboard, monitor, and mouse) such as the Raspberry Pi (http://raspberrypi.org) running an open-source Linux operating system can be purchased for less than $30 USD (Jolles 2021). Along with the decreasing cost of computers, there has also been a continually growing availability of microcontrollers, which are typically used to manage one or a few dedicated tasks with minimal software, as opposed to a multi-purpose computer with an operating system. Microcontrollers offer the ability to take analog voltage measurements or communicate with digital sensors, and can also be put into very low power modes when not actively sampling, allowing for battery-powered deployments that might last weeks or months. The Arduino system (http://arduino.cc) of low-cost microcontrollers and free open-source software is a widely used example of open-source hardware and software that is aimed at beginners and hobbyists. Additional initiatives, such as PlatformIO (https://platformio.org) provide software tools to make use of an even larger array of different microcontroller platforms.

Whether using a computer or microcontroller, the goal is typically to measure one or more physical phenomena using sensors, and to store or process those data, with the potential to also produce some physical output including user feedback or actuation of other devices (i.e., motors, lights, and so on). The sensing process involves transducing some physical characteristic (i.e., the distance between two shell valves, the movement of the heart, and temperature) into an electrical signal that can be registered and interpreted by the device.

Physical sensors can typically be divided into analog and digital sensors. Analog sensors produce a varying voltage signal in proportion to the physical characteristic they are sensing, and the computer or microcontroller must have the ability to measure and convert that voltage into a digital number that can be stored or manipulated, using an analog-to-digital convertor (ADC) that may be built into the microcontroller itself, or via a peripheral microchip. Digital sensors provide their own means to converting the physical phenomenon into a digital number, generally through an internal ADC. In some cases, modern digital sensors are capable of doing more processing of the signal, and/or making a more precise analog-to-digital conversion with reduced electrical noise compared to an ADC found on the microcontroller or computer. The microcontroller or computer then queries the digital sensor through a defined protocol to receive data or modify the digital sensor's settings. Common digital communications protocols include the Inter-Integrated Circuit (I2C) protocol, the Serial Peripheral Interface (SPI) protocol, or the OneWire protocol, in addition to several other protocols that are less relevant in the context of biological or environmental monitoring, such as the Closed Area Network (CAN) protocol. A particular digital sensor's datasheet will specify which of these protocols it uses to communicate, and the microcontroller or microcomputer can be set to communicate with that same protocol. Many analog and digital sensors can also be put into low-power sleep states or turned off entirely in between samples, and newer sensors designed for use with mobile battery-powered devices are often more aggressive about minimizing power usage compared to older sensor designs.

Commercial closed-platform and open-source hardware solutions both have the potential to interface with new sensors, but open-source solutions may provide the researcher with greater flexibility in some key ways. Device size and packaging can be modified in an open-source hardware workflow to suit the particular needs of a project, rather than relying on an existing commercial device that may not have an appropriate size, shape, or weight. Power usage might be minimized to a greater degree than existing devices, allowing the researcher to design smaller or longer-lasting devices. Costs may be reduced by being able to include only the minimal features needed for a project or using lower-specification parts, rather than relying on a commercial device's options. In instances where a project demands substantial numbers of replicate devices, lower hardware costs may also favor an open-source project, where funds for buying additional devices may be limited but researcher time for development and assembly is available.

The advantages of potential lower hardware costs and greater design flexibility must be balanced against the time and labor cost of building a custom device. There can be a substantial learning curve for some portions of the development and testing process depending on the existing skillset of the researcher, which might require the allotment of a substantial amount of time (weeks or months) to gain familiarity with the hardware and software design processes. However, a strength of the open-source hardware and software movement is the ability to make use of existing shared projects and off-the-shelf products to streamline some or all of the development process. On the hardware side, those shared projects might be existing electrical schematics, physical circuit board designs to be purchased and assembled by the user, or even fully assembled hardware available for purchase. On the software side, other users and vendors often share code that can be used to interface with various sensors and other peripheral devices (i.e., an onboard real time clock or microSD card), or even share fully functional programs that work with existing hardware devices and could be modified if needed. While in many cases ordering an existing commercial device may be more time-efficient, there may be cases where a researcher or student with a constrained financial budget, but available time to dedicate to learning these skills, may find that the open-source approach is the most feasible way to accomplish their research goals.

**Fig. 1:**
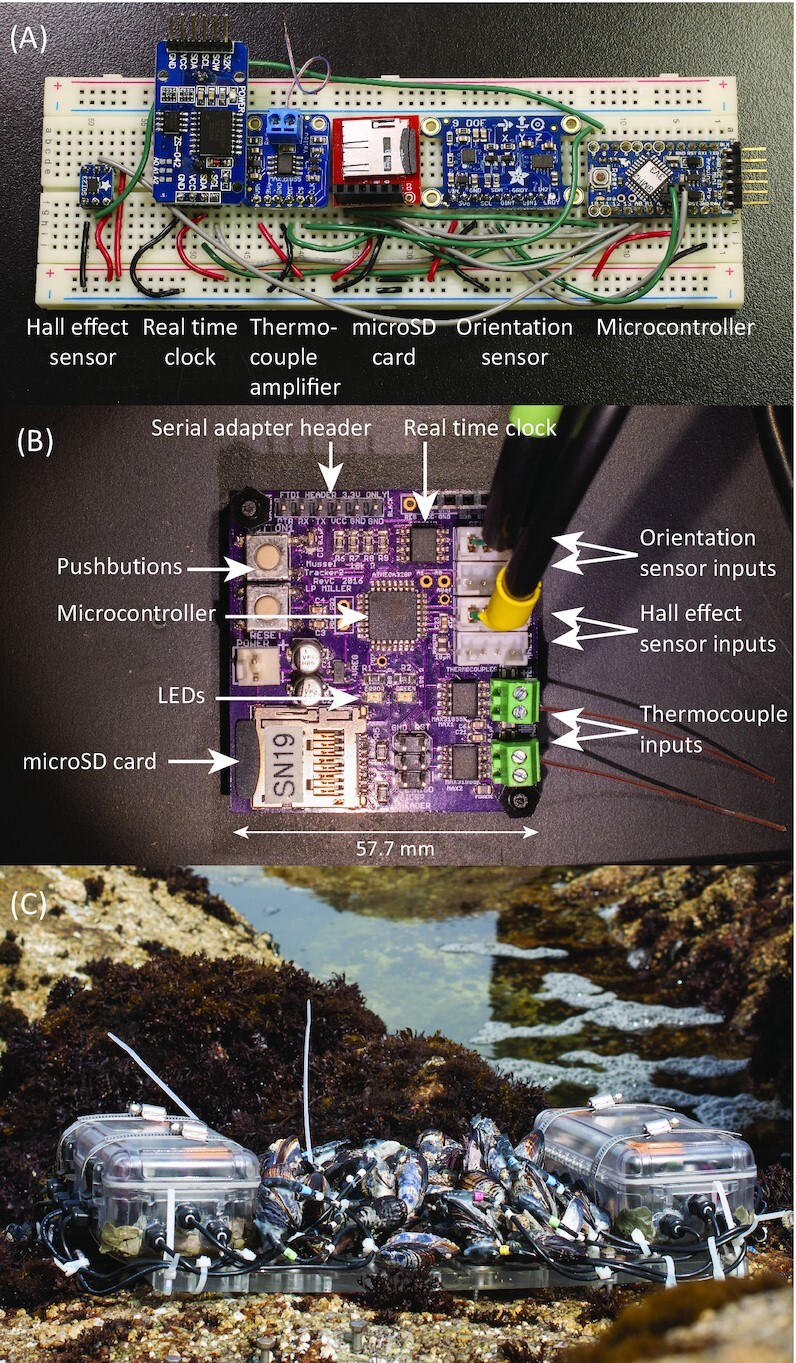
Example of MusselTracker data logger design (Miller and Dowd 2017). **(A)** Prototype MusselTracker assembled on a breadboard with a microcontroller, sensors, real time clock, and microSD card breakout boards wired together. **(B)** Final MusselTracker printed circuit board with primary components illustrated. **(C)** MusselTracker boards housed in waterproof boxes on the shoreline, with sensor cables attached to mussels.

**Fig. 2:**
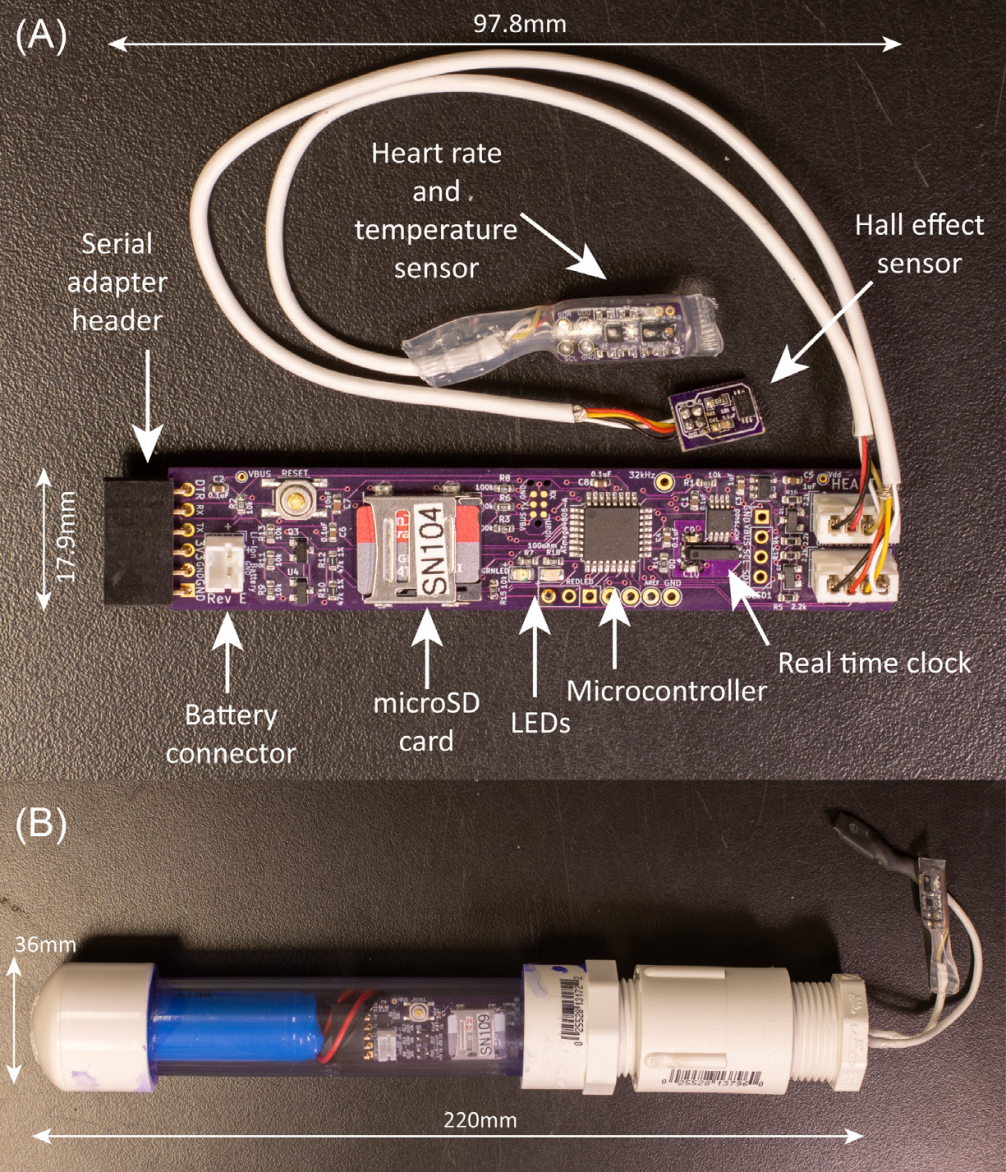
Example of the BivalveBit data logger. (A) Custom printed circuit board with microcontroller, microSD card, real time clock, serial adapter header, and attached sensor cables for heart rate, temperature, and valve gape measurement. (B) BivalveBit data logger housed in PVC pipe housing with lithium-ion battery.

Below, I describe a general workflow used for developing new data logging projects. I outline the process using two data logger projects. The MusselTracker (Miller and Dowd 2017) was designed to sample valve gape, body orientation, and internal body temperature for a pair of live mussels (Fig. 1 ). A more recent project, the BivalveBit (https://github.com/millerlp/BivalveBit), samples valve gape, heart rate, and shell temperature (Fig. 2). At the outset it should emphasized that there are multiple entry and exit points in this workflow, depending on the needs of the user and the availability of pre-existing designs, hardware products, and software. In many cases it may be sensible to start with a complete or nearly complete set of hardware that can be wired together and used with existing software. In other cases, it may be necessary to create a completely new design starting from the schematic phase in order to produce a device that fits the needs of the particular project.

## Methods for designing a custom data logging project

### Specifying project parameters and choosing components

Defining the parameters of the project should be the first step. Identify the organismal traits or environmental parameters that need to be measured and what setting the measurements will be made in, whether it is in the lab with constant access to a power source or in the field where battery power or solar power might be the primary options. For both the MusselTracker project and BivalveBit project, the goal was to deploy the devices in the intertidal zone where they would be submerged and exposed at low tide, so they would need to be powered by batteries and fit into watertight housings.

With the basic parameters outlined, appropriate sensors should be chosen, along with what kind of device (i.e., microcontroller) will be sampling those sensors. Given the vast array of available sensors on the market, choosing one may seem daunting if you are not simply using the same sensor as a previously published project. One option to speed the sensor choice process is to search for sensors available from commercial vendors that are already mounted on onto a small “breakout” board and accompanied by open-source software libraries designed to provide functions for communicating with the sensor. Purchasing a sensor mounted on a breakout board can simplify the wiring process during the prototyping phase, even if you later elect to use the same sensor on custom-made circuit boards.

For the MusselTracker project, I chose the three sensors based on different factors. For the Hall effect sensor, I identified a chip (A1395, Allegro Microsystems, Manchester, NH) that produces an analog voltage output, to be read by the onboard analog to digital convertor of the microcontroller. This particular chip was chosen because it included the ability to shut the Hall effect sensor off in between samples using an extra signal wire. Being able to put the Hall effect sensor into a low power mode was essential for prolonging battery life, particularly because Hall effect sensors tend to draw substantial amounts of current (3 mA during sampling for the A1395). Because we would only be sampling gape every few seconds or minutes, having a Hall effect sensor powered up continuously would lead to large amounts of wasted battery power. The goal would be to reduce the total current consumption of the system into the hundreds or even tens of microamps in between sampling events, so putting the Hall effect sensor into a low power mode was required. To reconstruct body orientation, I used a combination 3-axis accelerometer and 3-axis magnetometer. These sensors are commonly used in devices like mobile phones, so there are a number of similar chips on the market. I selected the LSM303D (STMicroelectronics, Geneva, Switzerland) because there was already a pre-existing breakout board and software available from an open-source vendor (Adafruit.com part number 1120, now superseded by new models), which could simplify the process of putting together a working prototype and take care of implementing the long list of software commands needed to communicate with the sensor (https://github.com/adafruit/Adafruit_LSM303). For measuring temperature with the MusselTracker, the goal was to use fine gauge thermocouple wire inserted through a hole drilled in the mussel shell to directly measure internal tissue temperature, so I wanted to use a chip that could convert the thermocouple voltage into a digital value and also provide a reference “ice point” temperature to convert the raw digital reading into a temperature. The MAX31855K thermocouple amplifier (Maxim Integrated, San Jose, CA) designed for use with standard type K thermocouple wire was also available on a convenient breakout board from Adafruit (part number 269), who also shared a software library to communicate with the chip (https://github.com/adafruit/Adafruit-MAX31855-library). For a data logging project, having the ability to record accurate time stamps was also important, so the MusselTracker design incorporated a DS3231M real time clock chip (Maxim Integrated) that could make use of an existing software library (https://github.com/adafruit/RTClib).

For the BivalveBit project, measuring heart rate was accomplished using an IR emitter/detector primarily designed for proximity sensing (VCNL4040, Vishay Intertechnology, Malvern, PA) that was available on a breakout board (Adafruit.com part number 4161) and had an associated software library (https://github.com/adafruit/Adafruit_VCNL4040). Because small thermocouple wire is relatively fragile and difficult to waterproof, I elected to use a semiconductor-based temperature sensing chip (TMP117, Texas Instruments, Dallas, TX) that could be attached to the outside of the shell surface adjacent to the heart rate sensor. The initial breakout board used for prototyping the design was purchased from SparkFun Electronics (https://sparkfun.com, part number 15805) and made use of their associated software library (https://github.com/sparkfun/SparkFun_TMP117_Arduino_Library). Valve gape was measured with the A1395 Hall effect sensor described earlier. Both the MusselTracker and BivalveBit devices make use of microSD cards to provide ample memory for sampled data. The task of communicating with the microSD card is simplified through the use of a dedicated library (https://github.com/greiman/SdFat) that allows comma-separated-value files (csv) to be written to the card. Those files can later be opened directly in common programs such as Microsoft Excel without requiring any specialized processing of the file beforehand.

Along with identifying the sensors, the user must decide on an appropriate microcontroller to run the device. When choosing a microcontroller, the most important characteristic is whether it has the ability to interface with the sensors that have been chosen, such as through an ADC or a particular digital communications protocol. As with choosing sensors, choosing a popular microcontroller with a large existing user base such as the Arduino system (https://arduino.cc) can improve the chances that the user will be able to find solutions to common problems and find example circuit schematics and code that can serve in the project. For the MusselTracker project, I based the design on an ATmega328P (Microchip Technology, Chandler, AZ), which is the same microcontroller chip used in the common Arduino Uno platform. This microcontroller includes an onboard ADC that could be used for reading the analog voltage from the Hall effect sensor. It also contains built-in peripherals to facilitate digital communications through the I2C, SPI, and UART (universal asynchronous receiver–transmitter) protocols, which would allow easy communication with the LSM303D accelerometer/magnetometer and DS3231M real time clock (via I2C), with the MAX31855 thermocouple amplifier and microSD card (through SPI), and with a regular computer through the UART serial system. Importantly for our purposes, the ATmega328P can be put into a low power sleep mode to reduce current consumption by at least an order of magnitude (from ∼10 to < 1 mA), and could be reawakened very quickly at regular intervals for sampling (four samples per second in our case, though sampling rates of thousands of times per second are easily achieved). The BivalveBit device makes use of a newer ATmega4808 microcontroller that provides more memory for program storage, while still maintaining compatibility with the Arduino software environment and add-on libraries via user-contributed software modifications (https://github.com/MCUdude/MegaCoreX) to the Arduino software.

### Building a prototype

Once the potential sensors and a microcontroller have been identified, the next phase should be creating a prototype circuit and software to run the device. Here again, leveraging designs and code from existing open-source projects can speed up the prototyping process. This might involve using exact copies of others’ circuits and software, or using those designs as guides to making one's own modified version. For example, the Arduino project publishes the full electrical schematics for all of their hardware products, which can provide insight into how the microcontroller should be wired up, along with other vital peripherals such as voltage regulators or crystal oscillators, and various small components such as resistors and capacitors that help run the microcontroller. Vendors of open-source hardware will similarly make electrical schematics available for their products (such as real time clock boards, SD card boards, and sensor breakout boards), while manufacturers of the individual sensor chips also produce datasheets that typically provide minimal working electrical schematics. If the sensors and other peripheral chips are available on breakout boards, prototyping using a common “breadboard” system can allow quick connection between the chips. Breadboards use a standard 2.54 mm (0.1 inch) grid spacing of holes that pins from a breakout board or microcontroller can be plugged into. Additional wire can then be inserted into the board to make interconnections between the pins of different components (i.e., a sensor and the microcontroller) without the work of soldering a permanent connection (Fig. 1A).

For the MusselTracker project, the initial prototypes were built using an Arduino Pro Mini, which houses an ATmega328P microcontroller in a form factor that plugs directly into a breadboard. The accelerometer/magnetometer and thermocouple amplifier were supplied on breadboard-compatible breakout boards, as were the real time clock and microSD card adapter. The Hall effect sensor was not directly available on a breakout board, so it was necessary to purchase a generic surface mount chip adapter board that provided soldering pads that matched the sensor's pin spacing and then spread those lines out to the 2.54-mm pin spacing of the breadboard. Short lengths of wire were used to interconnect appropriate pins on the microcontrollers and the sensors. For the BivalveBit project, prototyping was similarly carried out on a breadboard using an Arduino Nano Every (Arduino.cc part number ABX00028) which uses an ATmega4809 microcontroller, which is a variant of the ATmega4808 I ultimately used on the project.

For code to run the microcontroller and talk to the sensors and other peripheral systems, the open-source community shares a vast amount of example code. As a result, much of the development effort for the example projects detailed here could be focused on writing code to use the provided function calls to the various peripherals, rather than having to implement the low-level functions necessary to get data to and from the sensors and other peripherals. For the MusselTracker and BivalveBit projects, by the end of the initial prototyping phase I had a wiring design and software that were sufficient for collecting data from all three sensor types and saving them to the microSD card at the desired interval.

### Deciding to build a custom circuit board

For some projects, arriving at a working prototype built on a breadboard may be an exit point in this workflow if the prototype is sufficient for collecting data. In other cases, it may be desirable to continue on to making custom circuit boards. There are several cases where the investment in time to learn to use circuit board layout software and get custom circuit boards produced may be warranted. The use of push-in wiring on breadboards is susceptible to becoming disconnected due to handling, movement, or vibration. Breadboard prototypes are often susceptible to electrical noise from other sources, since the loose wires used to connect pins in the circuit may act as an antenna. For more complicated devices, the time required to wire connections on a breadboard, or to hand-wire and solder connections on “perfboard” (fiberglass board material with a perforated 2.54 mm grid of holes), may be substantial, so that making many replicate devices can be time consuming and fraught with the potential for incorrect wiring. Reducing costs may also be a motivation for designing custom circuit boards, because off-the-shelf microcontrollers and peripheral breakout boards may be relatively expensive and contain additional chips or features that are not necessary on the final design. For example, several of the sensors on breakout boards described earlier cost approximately $15 US, while the discrete sensor chips used on those breakout boards may cost just a few dollars, with further discounts for bulk purchases. For the MusselTracker data logger, the cost for purchasing all of the individual electronic components and manufacturing circuit boards came to $60 US per device (excluding batteries and housings), while the BivalveBit data loggers cost $24 US per device plus the additional cost of a battery ($10) and PVC housing parts ($8). In some cases the most important motivation may be the need for a custom size and shape that will allow the circuitry to fit into a certain size or shape of housing, which may not be possible with off-the-shelf devices. The MusselTracker device was destined to be housed in small waterproof boxes along with AA-size battery packs, with the goal of producing a low-profile housing that could withstand deployment on a wave swept rocky shore. The need to fit multiple MusselTracker boards in a small housing made a breadboard-based design less desirable, and the goal of minimizing power consumption also motivated our decision to build a custom printed circuit board (PCB) that only had the minimal number of chips on it to accomplish the data collection tasks. For the BivalveBit project, the design was intended to fit inside common nominal 3/4-inch schedule 40 PVC plumbing pipe along with an 18650-sized (18 mm diameter) lithium-ion battery for power, so that the entire assembly could be pushed into muddy estuary substrate leaving only the instrumented bivalve exposed on the surface. This constraint required a narrow circuit board width that was not available as an off-the-shelf product.

### Designing a custom circuit board

At the conclusion of the prototyping phase, there should be an electrical schematic based on the breadboard prototype's circuit design. Open-source software or free commercial closed-source Electronic Design Automation (EDA) software is used to transfer the electrical schematic into a format that can be used to design and build a custom PCB. KiCad (https://kicad.org) is a widely used free and open-source cross-platform PCB design software, while there are also several commercial PCB design packages that have free versions available to amateur and hobbyist designers. The commercial packages may come with limitations on the physical size of the PCB that can be designed, or contain other limitations such as the number of connections that can be made. Autodesk Eagle (Autodesk, San Rafael, CA) is a commercial package with a free version has been a common tool for many open-source hobbyists and vendors, and many open-source hardware projects make Eagle design files available. Some software, including KiCad, provides software tools to migrate design files from other programs such as Eagle, making it possible to utilize a large existing collection of shared hardware designs in a completely open-source workflow.

**Fig. 3:**
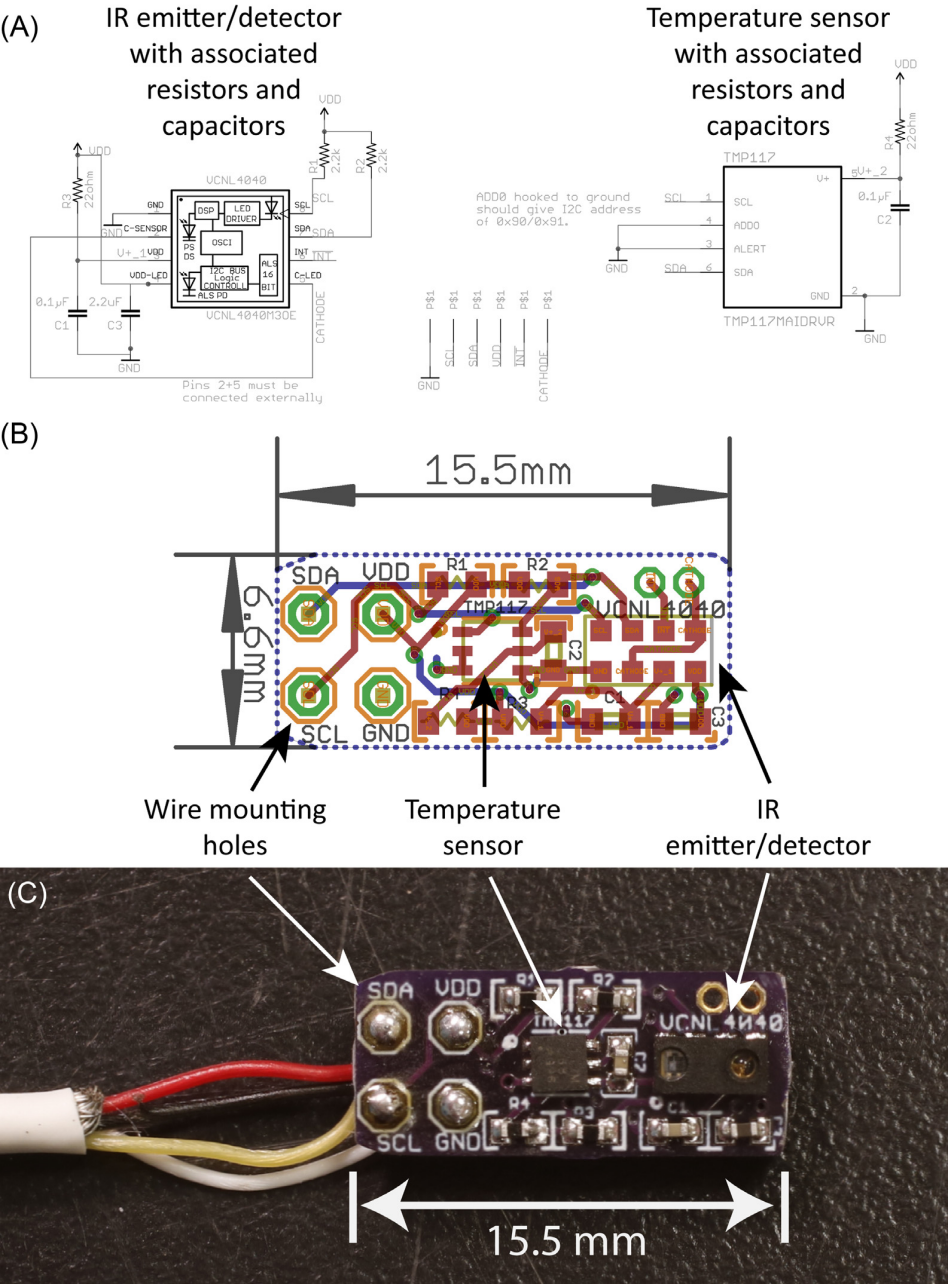
**(A)** Electrical schematic for a heartrate and temperature sensor board designed to be attached to a bivalve shell, created using EDA software. Representations of the primary chips (VCNL4040 IR emitter/detector and TMP117 temperature sensor) and associated components (resistors, capacitors, and wire mounting holes) were available for available for use from online sources. **(B)** PCB physical layout created in the EDA software. This file could be sent to PCB manufacturers for production in small batches. **(C)** Final assembled PCB with sensors, ancillary components, and data cable attached.

Designing a custom PCB starts with creating an electrical schematic of the device. Representations of individual chips with their individual pins are placed in the workspace, and those pins can be connected to other chips in the design to create the electrical schematic (Fig. 3A). The EDA software writers or community members make available representations of both specific chips and more generic items such as resistors or capacitors, so that the user can search for a particular chip and quickly add it to the design. With a complete electrical schematic put together, the same EDA software can then be used to create the physical layout of the circuit board, using representations of the physical “footprint” of each chip or device's pins that would be soldered onto the circuit board. The electrical schematic created beforehand contains the definitions of which footprint pins should be connected to others on the board, and the user then can manually route wire traces between those pins, or use the “autorouter” feature built into most EDA software. This step of the design process allows the user to define how big the PCB should be and where individual components should be placed. There are numerous online tutorials that walk through the steps of using EDA software (i.e., https://www.kicad.org/help/learning-resources/) so that a new user can learn the software relatively quickly.

This phase of the design process requires some consideration of how the device will be used and how it might fit into a housing, so that all of the necessary components of the device remain accessible during use. For example, header pins used for connecting a serial or debugging cable to a computer should be accessible, as should any removable memory cards, plugs, or buttons that might be used to interface with the device. For the MusselTracker PCB, I arranged the connections for the three sensor types to be located at one edge of the board. A reset button for the board and a header for connecting a serial communications cable were placed on the opposite edges of the board, so that they would not interfere with the sensor cables when the board was closed into the housing. The BivalveBit circuit board needed to be less than 18.5 mm wide (the inside diameter of the chosen PVC plumbing pipe) with low profile connectors and header pins to fit within the pipe.

For the sensors themselves, it may be desirable to have the sensor chip mounted on a separate small circuit board than can be attached to a wire cable and then placed precisely on the bivalve shell. Using the EDA software, I designed small individual circuit boards approximately 6 × 15 mm width and length that held the footprint for the appropriate sensor (Hall effect, IR heart rate, and accelerometer/magnetometer) and ancillary components (resistors and capacitors), and had holes for soldering wire cables that could run back to the main data logger circuit board (Fig. 3B and C).

### Producing and assembling custom circuit boards

Once a circuit board design has been created, there are numerous vendors that can use the native EDA software files (i.e., KiCad's .kicad_pcb file format or Eagle's .brd format) or the more standard Gerber file format produced by EDA software to create small batches of PCBs. Two examples aimed at hobbyists producing small quantities of boards are https://oshpark.com and https://seeedstudio.com. Boards are usually priced based on the size (square inches) and costs may range up to approximately $2 USD per square inch for a board with two-layer design (top and bottom layers where copper traces can be routed), with lower prices for bulk purchases. Production and shipping times may take roughly 2 weeks for standard service. There are a number of companies that will manufacture the circuit board and also offer assembly services, where they purchase the associated chips, solder the device together, and supply a fully assembled board. This process may be worth the additional cost in cases where you intend to produce many devices, particularly when balanced against time required to assemble and test boards by oneself. However, for the relatively small number of devices needed for my projects, I elected to assemble the boards in the lab. Many of the large resellers of electronic components will sell in small quantities to hobbyists and researchers, so the requisite chips and other components can be ordered without cost-prohibitive minimum order quantities (https://digikey.com, https://newark.com, https://mouser.com).

The prospect of soldering modern surface-mount electronics may appear daunting, but with a selection of appropriate hobbyist tools it is quite manageable. Surface-mount chips are meant to be soldered using solder paste and an oven rather than more traditional soldering iron and wire solder. Solder paste can be quickly spread over the appropriate locations on a PCB with the use of a low-cost stencil. Stencils can be produced from the same PCB design files or Gerber files used to produce the circuit board itself, and there are companies targeted at the hobbyist market that produce low-cost stencils (i.e., https://oshstencils.com). With solder paste applied to the circuit board, individual components can be placed with tweezers on the matching location of the board, and a standard stereo dissecting microscope found in many labs aids with manipulating small parts. To melt the solder paste, there are various common methods used by hobbyists, such as IR heater ovens designed specifically for soldering circuit boards, modified toaster ovens, electric hotplates, or “hot air rework” stations that let the user direct a steady flow of 230–270°C hot air at individual components to melt the solder paste. Soldering surface mount chips is possible with a high-quality traditional hand-held soldering iron, but using an oven or other method that can heat the entire board at once allows for a much quicker process when soldering many components.

After initial soldering, there is often the need to do some cleanup of solder joints where there might have been too much solder applied and solder bridges between pins form. In these cases, the soldering iron and a length of copper solder wick can be used to draw excess solder out of the joint. Here again, a stereo dissecting microscope will prove useful.

### Programming the device

With a commercial microcontroller development board like the Arduino Uno, the user can hook up a standard USB cable and communicate with the device to upload new programs or read out data over the serial connection. With a custom-built circuit board, the microcontroller chips shipped from the factory typically have no existing program (referred to as firmware when discussing microcontrollers) on them. This then requires the user to install their own firmware on their custom-built device. That device may lack a dedicated USB connection and instead rely on direct communication through the serial pins on the microcontroller using a USB to serial adapter chip (often referred to generically as a FTDI adapter or Serial adapter, available for $3–$15 from numerous sources). When working within the Arduino system, the standard method is to load a “bootloader” program onto the microcontroller once (commonly referred to as “burning” or “flashing” a bootloader), which then allows the use of the serial adapter connection to upload new revisions of the main firmware and to send serial data to and from a host computer. Burning a bootloader onto a new ATmega microcontroller built on the Arduino platform can be accomplished with a specialized programming board, available for $15–30 USD, or a standard Arduino Uno can be temporarily repurposed (see the tutorial Arduino As ISP, https://docs.arduino.cc/built-in-examples/arduino-isp/ArduinoISP). Once the bootloader program has been installed on the microcontroller chip, the USB serial adapter is typically used as the primary interface between the device and the user's computer.

Writing programs for microcontrollers is typically done using the C++ language, which the Arduino system uses for the user's main program and for contributed libraries shared by other users. Outside the Arduino system, other microcontroller platforms are programmed with C, C++, or a modification of the Python programming language known as CircuitPython. The large size of the Arduino community and large variety of shared libraries and example code make it particularly attractive for beginners, and many of the tutorials assume little to no prior electronics or programming knowledge.

## Housings and cabling

If the device you have built will be housed in the water or near the water, some form of water resistant housing will usually be a requirement for the project. Numerous manufacturers produce waterproof plastic cases that can be adapted for this purpose. For the MusselTracker project we elected to use commercial case purchased for about $30 USD, and used latches for easy opening and sealing. Because the sensors were mounted on cables that need to be run outside of the box, we used watertight bulkhead fittings that can be securely glued through a hole drilled in the wall of the box and then clamped around the sensor cable. Bulkhead fittings can be purchased to suit a range of cable diameters, and can be made of low-cost plastic ($0.50–$2 each) or more expensive and durable brass or stainless steel. Using bulkhead fittings allows for easier removal of damaged or leaking sensors.

For the BivalveBit project, the goal of producing many individual replicates for minimal cost led me to use household PVC plumbing pipe, which can be glued together permanently or connected with threaded fittings. Standard pipe thread sizes are tapered, so that as the two halves are screwed together they bind up and hopefully create a fluid-tight seal. In practice, it is often a good idea to use additional sealing methods such as pipe thread tape (polytetrafluoroethylene, Teflon® tape) applied to the threads before assembly and/or sealing with a layer of glue after assembly. Holes in the pipe housing where cables passed through can be sealed using polyurethane sealant (for example, Amazing GOOP, Eclectic Products, Eugene, OR), which was less expensive than bulkhead fittings and allows for the use of much smaller holes, with the downside of not allowing simple replacement of damaged or faulty sensors.

For cabling, I used 4-conductor 26- to 30-gauge cables, which includes standard USB (1.0 or 2.0) cables. USB cabling is available in bulk without cable ends installed, providing a low-cost source of multi-conductor wire. Wire with four conductors is sufficient to allow communication using the I2C protocol and UART serial protocol (requiring a voltage supply wire, a ground wire, and two data wires) and OneWire protocol (using one voltage supply line, one data line, and one ground line). For the analog Hall effect sensor used to measure valve gape for the MusselTracker and BivalveBit data loggers, four conductors allow for voltage supply, ground, the analog voltage output, and an input line to control the sleep function on the sensor.

Many common low-cost cables, including many USB cables, are jacketed in PVC material, which is seawater resistant and relatively flexible. In cases where abrasion on rocks or other surfaces may be a concern, I added an additional jacket of vinyl tubing or similar material over the exposed portions of the cable to act as a sacrificial layer. Sealing the small sensor circuit boards onto the cabling can be accomplished by potting the board and cable end in epoxy or glue, but I used adhesive-lined polyolefin heat shrink tubing for quick and clean assembly. As the heat shrink is shrunk with a heat gun, the adhesive lining flows around the cable and the circuit board, forming an effective seal. The distal end of the heat shrink tubing can be flattened with a tool while still hot to cause it to seal to itself. For the IR heart sensors, transparent adhesive-lined heat shrink can be used. The rubber compression fittings used in watertight bulkhead fittings seal well to PVC, and adhesives such as polyurethane glues can also be used to provide additional sealing around joints or seals on PVC-jacketed wires and polyolefin heat shrink tubing. For attaching the sensor to a bivalve shell, cyanoacrylate glue, in the thickened gel form, is suitable for initial attachment of sensors coated in polyolefin or PVC heat shrink, but additional adhesive in the form of two-part waterproof epoxy is recommended for long deployments in harsh conditions (A-788 Z-Spar Splash Zone Compound, Pettit Paint, Rockaway, NJ). An opaque layer of epoxy over IR heart rate sensors also helps to reduce extraneous light that can add noise to the IR detector signal.

Finally, the ability to communicate with a sensor and obtain samples from it does not necessarily guarantee that the sampled values will be accurate. With custom-built hardware, it is incumbent on the user to calibrate their sensors in order to ensure the data are trustworthy. For the MusselTracker device, we calibrated thermocouple temperature sensors using a high quality water bath and traceable temperature sensor as a reference. Gape sensors could be calibrated after installation on the bivalve shell, prior to deployment or after deployment when the animal had been sacrificed the but shell articulation remained intact. The accelerometer/magnetometer used to estimate body orientation required a thorough set of baseline measurements in all orientations in order to correct for the offset induced by the nearby gape magnet on the magnetometer and to account for the particular orientation of the sensor relative to the body of the mussel. With the IR heart sensor used on the BivalveBit, initial placement of the sensor required watching a live trace of the sensor output in order to see when the sensor generated the clearest signal. The IR sensor data are recorded as raw numbers and post-processed after deployment to extract heart rate estimates. Finally, the TMP117 semiconductor temperature sensor used with the BivalveBit device is factory calibrated, but it is still prudent to conduct checks of the temperature output against a well-calibrated reference temperature sensor.

## Discussion

In this paper, I have primarily focused on bivalve mollusc monitoring as a practical application of the open-source approach, but scientists have been applying these tools to a broad array of study organisms and their environments for many years (Jolles 2021). Examples include measuring forces on algae (Boller and Carrington 2006; Gandra et al. 2015), capturing terrestrial arthropods in pitfall traps (Buchholz 2009; McMunn 2017), tracking free flying birds (Shipley et al. 2017; Fahlbusch and Harrington 2019), monitoring egg turning behavior by birds (Shaffer et al. 2014), monitoring groundwater infiltration rates in caves (Beddows and Mallon 2018), measuring ocean wave height (Yurovsky and Dulov 2017; Lyman et al. 2020), tide cycling (Knight et al. 2021), and monitoring local-scale environmental conditions (Wickert 2014; Mickley et al. 2018). Open-source tools like the Arduino system have also been used to control aspects of experiments, such as controlling temperature treatments (Greenspan et al. 2016; Cocciardi et al. 2019; Baer et al. 2020), generating accurate tide changes in aquaria (Miller and Long 2015), and simulating saltwater intrusion into freshwater ponds (Lee et al. 2016).

The open-source hardware and software movements have created greater opportunity for researchers and practitioners to make use of automated data logging technology. It is possible to create a novel device from a blank starting page using open-source hardware design tools and open-source software packages, and create a customized device that can be tailored to the needs and constraints of the user's own study system. Using open-source tools can remove some of the costs associated with licensing proprietary software and perhaps obviate the need to use closed-source commercial equipment.

The open-source approach can streamline the process of developing a customized sensor solution. Leveraging existing shared design files can allow for rapid prototyping of simple or more complicated circuits, and making use of shared software libraries and tools can simplify the process of writing a custom program for one's own device. For an ever-expanding number of sensors, there is very little reason to reinvent the software tools from scratch when freely available software libraries might already exist for the individual sensor that has been chosen. For neophyte users, the large selection of tutorials and supportive communities such as the Arduino community can help lower the barrier to entry and flatten the learning curve to some degree.

The ability to build lower cost hardware can allow for greater replication while lowering the financial impact of lost, stolen, or malfunctioning equipment. The ability to build a device in the size and shape appropriate for a particular study system can make it more feasible to attempt new types of monitoring or experiments, in the laboratory and field. Science carried out with the open-source ethos applied to data sharing, but also hardware and software sharing, should help improve the ability to replicate published studies and expand into new lines of inquiry.

## Funding

The development of the MusselTracker device was supported by the National Science Foundation IOS [grant 1256186] to W.W. Dowd. The BivalveBit development was supported by grants to L.P.M. through the National Science Foundation [BIO-OCE 1904185], by the California State University Council on Ocean Affairs, Science & Technology (CSU COAST), California Sea Grant through the CSU COAST State Science Information Needs Program [award # COAST-SSINP-2021–003], and by the National Estuarine Research Reserve System Science Collaborative, which supports collaborative research that addresses coastal management problems important to the reserves. The Science Collaborative is funded by the National Oceanic and Atmospheric Administration and managed by the University of Michigan Water Center [NA19NOS4190058].

## Data Availability

Data and associated analysis code for the MusselTracker project are available from the Dryad Digital Repository (Miller and Dowd, 2017): https://doi.org/10.5061/dryad.2sd19. MusselTracker hardware designs and software are available at https://github.com/millerlp/MusselTracker and https://github.com/millerlp/MusselTrackerlib. Designs for the hardware and software of the BivalveBit data logger are available at https://github.com/millerlp/BivalveBit and https://github.com/millerlp/BivalveBit_lib.

## Notes

From the symposium “Open source solutions in experimental design’’ presented at the annual meeting of the Society for Integrative and Comparative Biology virtual annual meeting, January 3–February 28, 2022.
